# Neosalvarsan

**Published:** 1912-09

**Authors:** W. W. Quinton

**Affiliations:** Buffalo


### Neosalvarsan.

By MAJ. W. W. QUINTON, M.D.,
Buffalo.

THROUGH the courtesy of Victor Kœchl & Co., New York City, I have received recent German literature relating to the new product of the Ehrlich laboratories—Neosalvarsan.

The dosage recommended by Schreiber and quoted in the August number of the JOURNAL, was regarded by Ehrlich as being too liberal and Schreiber now agrees that small doses are to be preferred. Ehrlich's views on dosage have the support of Wechselmann of Berlin, Marschalko of Klausenberg, Weintraub of Weisbaden and Duhot of Brussels.

The average single dose for Men is .6-.75 Gm. Women, .45-.6 Gm. For older children, .15-.3 Gm. For nurselings, .05 Gm.

Maximum single dose:— Men, .9 Gm. Women, .75 Gm.

The treatment should begin with small doses and combined mercurial treatment as in the case of Salvarsan is recommended.

In early syphilis showing signs of disturbance of the nervous system, a short preliminary treatment with mercury is advised after which treatment with Neosalvarsan may be instituted using small doses varying from .15-.3 Gm. A second injection of the same amount is given and later injection of .4, .6, .75 and as a maximum .9 Gm.

In early chancre of at most 14 days duration with negative Wassermann and slight involvment of the lymphatics, one may use to advantage a stronger initial dose.

In cerebral lues or meningitis the beginning dosage must be small.

Subcutaneous injection must be avoided under all circumstances. For intravenous injections .15 Gm. Neosalvarsan is dissolved in 25 ccm of freshly distilled water. For intra-muscular injection:—I Gm. of Neosalvarsan dissolved in 22 ccm of distilled wated will give an isotonic solution. Therefore 3 ccm of distilled water should be used to dissolve .15 Gm. of Neosalvarsan. The injection is best made with a sterile 4% saline solution using chemically pure NaCl and freshly prepared water. It must be at room temperature (20-22C) and must be injected immediately. Under no circumstances must the solution be warmed.

